# Clinicians’ experiences of becoming a clinical manager: a qualitative study

**DOI:** 10.1186/1472-6963-12-421

**Published:** 2012-11-22

**Authors:** Ivan Spehar, Jan C Frich, Lars Erik Kjekshus

**Affiliations:** 1 Department of Health Management and Health Economics, Institute of Health and Society, University of Oslo, PO Box 1089, Oslo, NO-0318, Norway

**Keywords:** Leadership, Administration and organization, Health services administration, Nurse manager, Doctor, Qualitative research

## Abstract

**Background:**

There has been an increased interest in recruiting health professionals with a clinical background to management positions in health care. We know little about the factors that influence individuals’ decisions to engage in management. The aim of this study is to explore clinicians’ journeys towards management positions in hospitals, in order to identify potential drivers and barriers to management recruitment and development.

**Methods:**

We did a qualitative study which included in-depth interviews with 30 clinicians in middle and first-line management positions in Norwegian hospitals. In addition, participant observation was conducted with 20 of the participants. The informants were recruited from medical and surgical departments, and most had professional backgrounds as medical doctors or nurses. Interviews were analyzed by systemic text condensation.

**Results:**

We found that there were three phases in clinicians’ journey into management; the development of leadership awareness, taking on the manager role and the experience of entering management. Participants’ experiences suggest that there are different journeys into management, in which both external and internal pressure emerged as a recurrent theme. They had not anticipated a career in clinical management, and experienced that they had been persuaded to take the position. Being thrown into the position, without being sufficiently prepared for the task, was a common experience among participants. Being left to themselves, they had to learn management “on the fly”. Some were frustrated in their role due to increasing administrative workloads, without being able to delegate work effectively.

**Conclusions:**

Path dependency and social pressure seems to influence clinicians’ decisions to enter into management positions. Hospital organizations should formalize pathways into management, in order to identify, attract, and retain the most qualified talents. Top managers should make sure that necessary support functions are available locally, especially for early stage clinician managers.

## Background

Challenges with managing patients with complex chronic diseases, advanced and expensive treatments, and growing societal expectations to the health care system, have raised the awareness of effectiveness and quality of care [1,2]. In addition, a focus on clinical governance [3] requires “a model which recognizes clinicians' central role in the design, provision, and improvement of care” [4]. There has consequently been an increased interest in recruiting, developing and encouraging clinicians to take on management positions in health care [1,4-8]. International research initiatives have recently been formed, including the European Cooperation in Science and Technology Action: “Enhancing the role of medicine in the management of European Health Systems” [9]. In addition, a range of leadership development programs have been launched in the NHS, including The Clinical Leadership Competency Framework project [10] and The Medical Leadership Competency Framework [11]. In Irish hospitals, the integration of clinicians into managerial roles has been recognized “as a key determinant of operational effectiveness” [12]. The focus on clinicians in management is not limited to Europe, but is seen internationally, including in countries such as Australia [5] and New Zealand [13].

The involvement of clinicians in management has received interest also in Norway, following the introduction of unitary management through the Specialist Health Services Act in 2001. Recommendations from the Office of the Auditor General of Norway state that clinicians should become more involved in budgetary and strategic decisions, in order to improve the economic efficiency of healthcare organizations [14].

Understanding factors that influence management development in healthcare organizations is crucial for creating environments in which clinicians can develop the skills and expertise needed to become successful managers [15]. However, little is known about the factors that influence individual clinicians´ decisions to become managers, and how they experience the transition from clinician to manager. The aim of our study is to explore clinicians’ experiences of becoming managers, in order to identify drivers and barriers to recruitment and development of clinical managers in hospitals.

Leadership can be enacted with and without formal authority [16]. While leadership is often understood as motivating or influencing others to produce change, management is usually described as achieving specific results by planning, organizing and problem solving [17]. Many authors have used these terms interchangeably, as both activities are usually integrated in formal management positions [18]. In order to avoid conceptual confusion, we refer to clinical managers as clinicians in formal management positions who may or may not retain a role in clinical work. This differs from the term “clinical leadership”, which is an often used term in the NHS [10]. Reaching a consensual definition of clinical leadership has proven to be difficult [19]. Edmonstone [20] refers to clinical leaders as someone who retains a clinical role while also engaging in management related activities, such as strategic and collaborative work with health care managers and professionals. This definition excludes clinicians who have become full-time general managers in hospitals and other health care organizations. Such managers, however, are included in our own definition.

### Theoretical framework

The literature on management and recruitment can broadly be differentiated between sociological theories of professions and the more “generic” management literature, often applied to the private sector. The former perspective has emphasized professional dominance and autonomy as underlying motives for engaging in management [21-23]. According to the sociological perspective, clinicians are motivated to seek and maintain influential positions, as their profession is engaged in a struggle for dominance and self-governance against competitive forces. These forces include the competing logics of market forces and government regulations [21], as well as other professions competing to expand and maintain their jurisdictions [22]. Studies of doctors in management positions tend to lend support to this perspective. For instance, Doolin [13] found that many doctors in New Zealand hospitals chose to enter management in order to protect medical practice from interventions by general managers. Forbes, Hallier, & Kelly [24] interviewed Scottish doctors that had recently engaged in management positions, and found that many had assumed management roles in order to protect their specialties from outside influence or from individuals they considered to be inappropriate clinician-managers. Similar accounts have been gathered from interviews with Norwegian doctors in management [25,26]. Edmonstone [19] points to the traditions in medicine of a representative, rather than hierarchical function in management. Doctors are accountable to management, but also to their peers, who continue to regard them as representatives of their own views and interests.

While most scholars in the sociological tradition have focused on doctors, similar ideas can be extended to other clinical professions, including nurses. Norway is one of the few countries where reforms have seen nurses competing directly with doctors for management positions. According to Johansen & Gjerberg [26], Norwegian nurses have assumed management positions in order to increase their professional recognition and status. This explanation is in accordance with sociological theories about professionalism, in which management positions become instrumental in strengthening one´s own profession. Professionalism is in sharp contrast to how managers have been depicted in the more “generic” management literature, where managers are described as individuals who seek towards management out of intrinsic motivation:

“The management models in the private sector highlight characteristics like innovativeness, creativeness and competency in management. In addition, managers are expected to show a spirit of entrepreneurship, high motivation and responsibility. Ideal manager type is one who has visions, leads via ideas and example, and strives towards a goal” [27].

Similar attributes have been emphasized in the New Public Management doctrine [28], which portrays the ideal manager as a person who is responsible [29] and passionate about management, and who is committed to the interests of the organization. These characteristics have been endorsed by policy makers within the NHS [10], who are eager to involve motivated clinicians, serving as “model managers who are committed to meet the requirements of the new public management” [27].

**Figure 1:**
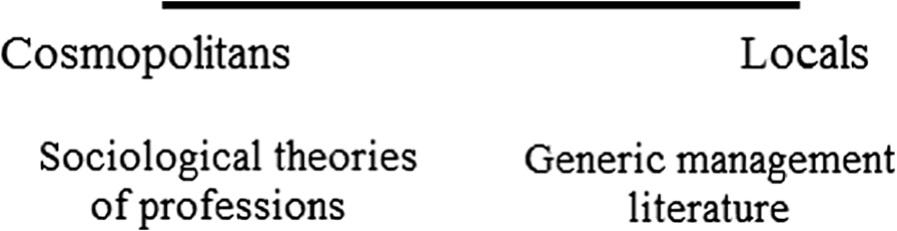
The two predominant perspectives on management, placed on a continuum of organizational identity and commitment.

Although the two perspectives differ in terms of the underlying motives for engaging in management, there appears to be an underlying assumption of voluntariness. While the former describes engagement from a strategic standpoint, the latter suggests that individuals seek management positions out of interest and motivation for the task. According to Gouldner [30], there are two ideal types of latent identities in an organization: “cosmopolitans” and “locals”. Cosmopolitans are characterized by a strong commitment to professional values and skills, a strong outer reference group, and weak loyalty towards the organization. Locals tend to be less committed to professional skills, have a local reference group (such as managers in similar positions within the same hospital), and show stronger loyalty towards the organization. While sociological theories tend to emphasize professionals as “cosmopolitans”, general management theories tend to view managers as “locals” [10,27]. The two perspectives might therefore be ordered along a continuum, from a more cosmopolitan identification in the former, to a more local identification in the latter, as illustrated in Figure 1. The figure suggests that different aspects must be taken into account when recruiting and developing managers in the different sectors. We seek to further expand the knowledge in this area of research. 

## Methods

### Participants

We found a qualitative approach suitable, and recruited 30 clinical managers for interviews. The managers were chosen from clinical departments and sections within two Norwegian health trusts. We used a maximum variation sampling strategy in order to include a wide range of informants and collect a broad range of experiences [31]. We recruited the participants through their superiors in the organization. One clinician declined to participate. The sample includes 16 nurses, 13 doctors, and one participant with another healthcare background. Mean age was 51 years (ranging from 36–65 years). While some of the participants had held their position for several years, others had only held their position for a few months. Characteristics of participants are presented in Table 1. Twenty of the managers were recruited from medical departments, and ten were recruited from surgical departments. In total, they spanned across four geographical locations (hospitals) in two health trusts. One health trust had a five-level hierarchical management structure (executive director, division management, department management, section management and unit management), while the other had four management levels. 

**Table 1:** Characteristics of participants (N = 30)

**Characteristics**	**No**	**%**
**Gender**		
Female	17	(57)
Male	13	(43)
**Age**		
36-45	9	(30)
46-55	12	(40)
56-65	9	(30)
**Management level**		
Department	17	(57)
Section (includes nine first-line managers)	13	(43)
**Mean age**		
Doctors	55	
Nurses	49	
Other clinical background	40	

### Data collection procedure

An interview guide was developed on the basis of existing literature and two focus group interviews with a total of 20 clinicians who participated in a management course. The first author conducted tape-recorded, face to face in-depth interviews with 30 participants. The interviews were done at their workplace. The participants were, among other aspects of being a clinical manager, asked about their career paths towards their current management positions. The interviews lasted from 45 to 90 minutes. The first author also observed 20 of the participants in management and staff meetings and during informal talks with colleagues. In interview studies, realities are constructed from respondents´ languages, based on how they conceptualize practices and experiences. Observations enable the researcher to generate a (partially) independent view of the same experiences which respondents draw on to construct their realities [32]. Combining observations and interviews may therefore provide different data on a given phenomenon. We wanted to investigate whether observations would validate, expand on, or contradict accounts given in interviews. A special interest was on the barriers and facilitators in the manager role. Observations were carried out on the same day as the interviews. The author usually met up with participants at the start of their working day, and followed the participant throughout the day. One participant was followed over the course of two consecutive days. The author did not participate in clinical consultations with patients. Field notes from the observations were written down and kept for later analysis.

### Data analysis

Several steps were performed to ensure the quality of the analysis [33]. Firstly, NVivo8 computer software was used to facilitate the analysis of interview transcripts. The interviews were analyzed by systemic text condensation, according to the principles of Giorgi's [34] phenomenological analysis. The analysis followed four steps: (1) Reading all the material to form an overall impression; (2) identifying units of meaning representing different aspects of the participant´s career paths and subsequently coding for these units; (3) condensing and summarizing the contents of each of the coded groups; and (4) generalizing the description and contents reflecting participants´ management paths and motivations. In addition, transcripts of several of the interviews were analyzed for content and structure by all three authors of this paper, resulting in general agreement on a coding frame. Lastly, citations were translated to English by an experienced translator and then back-translated to Norwegian by the authors. This was done to detect possible semantic differences between the translated and original versions. Field notes from observations were analyzed for emerging themes, independently from interviews. Relevant themes were then assessed against findings from interviews, with special interest on observations that could illuminate accounts which participants gave in interviews.

### Ethical considerations

Ethical approval to conduct the study was granted by the Norwegian Social Science Data Services (ref: 23228/2/LT). Written consent to participate in the study was obtained from all study participants.

## Results

### Developing leadership awareness

Many participants recounted personal characteristics that they believed had predisposed them towards entering management. A female surgeon suggested that she had inherited a natural authority from her father:

“[My father] has always been a leader. And his father was a foreman […] I believe that personalities are inherited”.

When reflecting on their careers, participants described that they had always been taking responsibility, such as taking on commissions from a young age and becoming elective representatives. They speculated that this might have prompted them to seek or accept managerial proposals, although they had not initially envisioned any career plans involving management. Other explanations were centered around being outspoken or informal leaders. This is reflected in a statement from a male surgeon, who stated: *“I have become a manager because I cannot shut up”.* Some participants, mostly notably at the department level, described themselves as energetic and inclined towards seeking new challenges. One doctor cited boredom in his job as a clinician, as his work had become characterized by routine after years of experience in clinical practice:

”It becomes unsatisfying, that is a reason that I am sitting here now. […] I could perform it so well and felt so confident […] then the work day also becomes sad, kind of boring”.

He described how he had become easily bored in other situations too:

“I attempted several years ago to teach at the medical school [..] And I remember the first group of students, they were very interesting students. I used evening after evening to make nice [lecture] slides… The next group, it was ok […] and the third time I had had it up to my throat. I couldn´t be bothered to say the same things for the third time… and that probably characterizes me somewhat. I… need to have changes”.

Some participants recounted that they had a need for controlling their surroundings, while others admitted that they liked the feeling of power and being able to influence decisions. A male nurse said that he had wanted to influence decisions, but that he was not able to do this as a nurse. He said that this had sparked a desire to become a manager instead:

“I have enjoyed working in teams, but never [when] someone else has decided many things for me… and that corresponds very badly with the nursing profession […] And the need I had, contrasted with always being overridden by a professional group [doctors] with knowledge, power and authority, that suits me badly”.

### Taking on the manager role

With the exception of some clinicians who actively sought the position because they wanted to seek a new and interesting challenge, or promote professional interests, many of the initial entries into management were characterized by informal ways of recruitment, often by persuasion from the current manager. Participants stated that they had not had ambitions of becoming a manager initially, but that their superior, who was either retiring from work or stepping down, persuaded them to take their place. This was a consistent account given by the participants when describing their first, and sometimes consecutive manager roles. Participants expressed a feeling of pressure following these encouragements, which drove them to apply for the position. They recounted that they knew they would effectively become the new manager, as there were rarely other applicants for the position. Some participants had to take some time to think over the offer before accepting it, because it came suddenly and unexpectedly. One nurse was unsure whether to take on the job as a department manager, because she had no experience or preparation for the role. For her, the confidence in the skills of the staff was a contributing factor for finally accepting the management proposal:

“The reason why I dared was that there were so many competent people in that department, so I thought it could not possibly be hard to be the leader of the flock here, because there is so much competence”.

A doctor who also had to take some time to think through the offer, finally accepted the proposal because of a matter of principle, as he did not want the job out of personal interest or motivation:

“But I have the view that I think it is important that also doctors are managers. Not that all managers should be doctors, but that at least some managers are doctors. […] And then I was going around thinking that if I mean that, then perhaps I have to take the consequence of that view, and then at least be a manager for a while”.

Some participants experienced a pressure to accept the management proposal due to choices they had made in the past. A nurse had taken a course in management and team building at a business school, in order to increase her managerial competence after being asked to take over as a manager assistant at her section. Shortly after, she received a phone call from her supervisor who strongly urged her to take a section management position that had opened up. Although she wanted to take some time off after finishing the course, she was eventually persuaded to take the position:

Interviewer: “What were you thinking when you received the phone call?”

*Participant: “I thought that I really didn´t want to […] but I have to admit that my current boss has quite strong persuasiveness and challenged me strongly about the fact that I had gone to the business school. [My boss] said `youdo[emphasized by the participant] mean something by that?´”*

Another nurse who worked as a manager assistant was formally and automatically appointed as the section manager, after the previous manager had stepped down. Some of the participants with a medical background mentioned that the motivation to protect their own profession from external influence had pressured or driven them into taking a management position:

“Advancing the professional field was the reason for why I applied. One could say that it was kind of a negative motivation, that I saw that it wasn´t so many others that were appropriate or… more suitable to do it, myself I am perhaps more of a professional man and engaged with the profession and research, so that I believed I could contribute to preserve and develop the profession in the hospital”.

A surgeon used the words “painted myself into a corner”, when describing an attempt to prevent someone else from being chosen for a vacant management position:

“The person they were about to hire was someone I could not live with as a boss, and the others in the department could not live with as a boss either […] so I went into dialogue with the management and painted myself in a corner, where finally the only solution was that I applied for the position as department manager, something I really hadn´t planned”.

Only one participant was actively recruited to her current position as part of a formalized system, in which nurses took turns holding a section management position for a year.

### The experience of entering management

Participants experienced that they had been “thrown into” the management position, and that they were unprepared for several aspects of their new position. The most significant challenges were related to the workload and understanding the language and procedures associated with budgets and HSE (health, safety and environment). Some experienced the job as lonely and wished for a mentor or colleagues with whom they could share experiences. They had a sense of being left to themselves, having to *”reinvent the gunpowder”* or learn management *“on the fly”.* One department manager with a medical background told that he longed for a book with “the right answers”, which he could go to when dealing with medical issues:

“We don´t have, where is the book, you know. And I can go to my book if I receive a professional question, so that I can find out what is recommended and the reference list for those recommendations and so forth. And if I bother, I can even go in and read it myself, and see if I agree, you know? And that reference list does not exist here”.

A consequence of the unpreparedness was that participants saw their days being filled with increasing workloads. Participants told that they did not have time to perform their managerial tasks in a satisfactory way, and many had a long list of unread emails. One manager had a list of several hundred unread emails. A nurse said that the unattended workload had become so large after she returned from a vacation that she *“contemplated a new vacation in order to escape the workload*”. A doctor who was appointed as acting manager when the previous manager went into a year-long management course, had difficulties completing tasks in time, because he continuously had to learn new terminology associated with each new task. The number of new tasks increased faster than he was able to finish the old ones. The lack of local support personnel, such as IT support and financial controllers, was also mentioned in the interviews, and several participants wished for an assistant that could relieve them of administrative work. One of the managers had to assemble new office furniture by herself. Some participants told that the lack of organizational support prevented them for practicing newly learned skills that they had acquired from external management programs; they were to busy with administrative tasks. One participant said that he experienced increased interest in management after having participated in a national top management program. The interest waned quickly after returning to work, because administrative work had piled up in the meantime. Some of the managers at the larger departments had a formal assistant, and while one of the managers described this person as *”indispensable”,* because the assistant categorized her emails in a prioritized order, another said that his assistant had made the job *“ more livable”*.

Participants mentioned that their motivation for taking the manager role was not related to financial incentives, but doctors told that they now worked more than they had done as full-time clinicians, for less money. A recurrent theme was that the payment inherent in the manager role did not compensate for the ensuing overwork. Doctors had reduced the amount of time they spent on patients, but they usually retained some degree of clinical work.

Another theme that emerged in the interviews was the challenges related to task delegation. Some participants were unsure of what tasks they could or should delegate. A clinician who had been three months in a new department manager position felt that this affected his job satisfaction negatively:

*“I think that this istoo[emphasized by the participant] much, too much that I haven´t managed to delegate yet, because everything is new and unknown and there are too many barriers for this to be a gratifying job now. Unfortunately, I don’t really enjoy being in this role”.*

Others wanted to maintain an overview of every aspect of the organization, including personally overseeing as many emails and assignments as possible. One manager had taken on so many tasks that she was unsure if she wanted to continue in management:

“Because I do feel somehow that I have become stuck, that there are many assignments, and I think many things are exciting, maybe saying yes to too many things, assignments have become pretty extensive […] it has become pretty all-encompassing”.

A third group felt guilty for burdening their assistants or managers below in the hierarchy, because they were already overwhelmed with work. One participant felt that other managers were delegating too much:

“And then we have manager assistants that can take something of course, but, it´s about how much you want to delegate to them. And some people… I feel that some people are maybe delegating too much to them”.

While the stories above were characteristic of participants who were new in the role, participants with more experience were under the impression that a good manager was someone who delegated work tasks, rather than attempting to do everything themselves. However, participants also emphasized that, unlike in private companies, they were not allowed to hire their own support personnel.

Finally, some of the nurses who had become department managers experienced resistance from medical staff. Encouragement and support from colleagues was recounted as important in the process of overcoming this resistance and learning to take unpopular decisions, as illustrated below:

“So in the beginning when I first took over as department manager, I felt that everybody at times were against me, I won´t forget that once […] everyone was angry, and then one of the doctors I know came in [to my office]… and then I cried. And then she says: `now they are tough on you´. `Yes´, I said, `now they are so tough on me that I don´t know if I can bear to be in this situation any longer´. And then she said: `remember that you were not chosen in this position so that you would be liked, you are here to do a job, and that´s why you are here´. And that´s true, it´s okay to think about that now and then”.

Observations validated the accounts that participants gave about their experiences of the manager role. Observations confirmed that clinicians struggled with terminology related to finance and health, safety and environment. There were also examples of participants receiving urgent emails and work tasks during the day which meant that other planned tasks needed to be postponed, simultaneously increasing their total workload. In one specific case, a department manager struggled to delegate work tasks to his section managers during a management meeting, because of reluctance on the latters´ part to take on the task. In the end, the issue was left unresolved, because no one volunteered or accepted to do the task. Accounts that participants gave in interviews were also repeated in discussions with other healthcare workers and colleagues, indicating consistency in attitudes.

## Discussion

We found that there were three phases in clinicians’ journey into management; the development of leadership awareness, taking on the manager role and the experience of entering management. Participants had not anticipated a career in clinical management, and experienced that they had been persuaded to take the position. Being thrown into the position, without being sufficiently prepared for the task, was a common experience among participants. Being left to themselves, they had to learn management “on the fly”. Some were frustrated in their role due to increasing administrative workloads, without being able to delegate work effectively.

### Path dependency

A recurrent theme from the interviews was the experience of pressure towards taking a management position, in which some clinicians became “trapped” or restricted to a specific path. This was mentioned both by clinicians who sought management training to function better in their new management role, and by doctors who wanted to protect their professional interests. Following the descriptions above, we find the concept of path dependency relevant for illustrating our findings. The path dependency literature emphasizes that “history matters” [35], as actors are often tied to previous decisions which are hard to reverse [36]. The literature has mostly been applied to a macro level of analysis, such as describing the variations in national health service reforms [36,37]. Our findings suggest that the idea of path dependency could be applied also at the micro level, in which an initial decision to enter management can tie the clinician to an existing path, and close off other paths due to internal or external pressures. Although other authors have found instances of doctors becoming managers by “accident” [24,38], we find that the same can apply for nurses. Furthermore, the concept of path dependency suggests the process by which some clinicians “accidentally” enter and stay in management, or in extreme cases might become “stuck managers” [38]. Drummond and Chell [39] have applied the term “entrapment” in describing the career decisions of some lawyers. They described a process where individuals make decisions to take promotion for economic reasons. When later regretting their choice, they could not find a way to get back to doing the work they liked. Clinicians do have the option to opt out of a management role and return to clinical practice. But there also appears to be a belief among clinical managers of a “point of no return” [40] in abandoning clinical work, which leaves the clinician with few options but to pursue a full-time management career.

**Figure 2:**
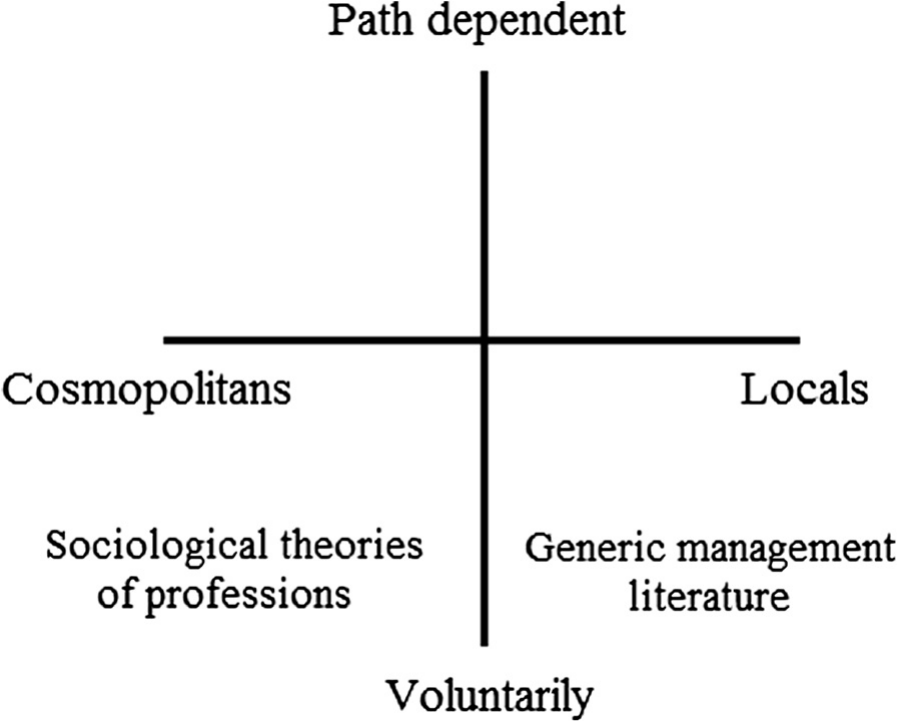
Contrasting perspectives on clinicians´ engagement in management.

Our study further suggests that the full range of motivations for entering and sustaining formal management positions are not easily captured by either sociological theories of professions or general management theories. Firstly, the notion of professional actors pursuing management positions in order to secure autonomy [21,22] was shown to have limitations in accounting for the management motives given by our participants. Although some doctors stated that they were motivated to protect their work from outside influence, other participants came into management reluctantly and for other reasons. And although some were drawn to new challenges, few of the participants in our study recounted strong initial ambitions towards engaging in management. Indeed, participants in our study were generally less enthusiastic towards engaging in management than what could be expected from the generic management literature. The emergence of doctors and nurses in management positions could evidently be more path dependent than what is implied by existing theories. Figure 2 expands on the illustration we presented earlier in the paper. Our results suggest that an added dimension is needed to fully grasp the processes by which clinicians become formal managers. While the concept of path dependency runs counter to the implicit notions of the generic management literature, it does not necessarily dismiss the professional perspective. Rather, it could serve to expand the understanding of how clinicians who are professionally invested become and remain managers. 

We do not dismiss the idea of clinicians situated in the lower right quadrant of our figure. Indeed, some of our participants recounted more positive journeys into management, as have clinicians in other studies. Mo [25] has given an account of Norwegian doctors in department management positions and their motivations for entering management. Although some felt persuaded to apply, others had entered management because of a feeling of curiosity. Forbes and colleagues [24] have noted that some doctors could be described as “investors”, actively pursuing management as an alternative to medicine. Although we find reason to question the precision of current theories in explaining the motives of clinicians in management, these theories could be more precise when applied to upper management positions, where status, pay and competition for positions is higher. Chief executives in a study by Ham et al. [41] displayed mostly positive attitudes towards their job. Hoff [42] also found that doctors in upper-level management reported greater job involvement than doctors at lower levels of management.

### Being unprepared for the management role

There were similarities in how clinicians experienced the first meeting with their new position. Many were unprepared for the challenges in their new role and struggled with increasing workloads and the lack of organizational support. In this regard, the accounts from our participants somewhat mirror that of doctors who become chief executives in the NHS, in that both groups received little structured advice and guidance as they transitioned into management roles [41]. Some of our participants noted that they had to learn management “on the fly”, and one doctor longed for a form of reference guide. Entering into management is a significant transition from clinical work, where tasks and routines are usually standardized, and in some way offer ”a relative oasis of calm and predictability” [41] compared to the dynamic tasks inherent in a management role. Although some of our participants mentioned management training as part of their route into management, it is possible to assume that clinicians who enter management through path dependent routes might be less prepared for the inherent challenges in the new position. A consequence could therefore be that they are more likely to experience the new position as overwhelming. In addition, increasing workloads may follow from an inability to delegate sufficiently. Lord and Hall [43] studied the development of leadership skills ranging from a novice to expert level. The authors concluded that “early attempts at leadership are guided by leaders´ desires to match their surface features (e.g., behaviors) to implicit theories of effective leadership”. It appeared that participants in our study initially sought to retain an overview of the whole organization. Clinicians with longer experience in managing were more likely to mention that they had learned to organize effectively, and that they had to derive support from individuals with different expertise from themselves. Chief executives in Ham´s study [41] also recounted that they had learned to involve colleagues in supporting roles:

“A key theme here was the importance of recognizing gaps in competence and experience that needed to be filled by others. This had often resulted in the appointment of experienced colleagues as chief operating officers, medical directors and other roles to ensure that appropriate support was available” [42].

Kane-Urrabazo [44] notes that delegating tasks is among a healthcare manager´s central responsibilities. Healthcare professionals in the NHS who received a three-day course designed to examine their own behavior as managers, reported that they took on less responsibilities and delegated more to their staff [45]. This suggests that experience alone is not the only prerequisite for improving management skills, and that effective delegation might be learned at an earlier stage than what is currently the case.

### Methodological considerations

We were able to combine insights from both interviews and observations to strengthen our findings and insights. Observations of participants in dialogue with other staff members confirmed accounts that they had previously given in interviews. The study was done in a Norwegian hospital setting, but we believe that our findings are transferable to other countries without explicit policies and systems for recruiting and developing clinical managers. The accounts given by the participants in our study should be understood in light of a Scandinavian context where cultural norms against showcasing or boasting may be present, a prevailing social code in Nordic countries [46], which Gullestad [47] calls equality based on conformity. These cultural norms may perhaps account for why we did not find more obvious “investors” [24] among the participants, describing themselves as natural or “born” leaders and innovators. Another limitation is that we did not seek to include clinicians that had either left management or turned down such offers. By including such participants, we could have been better able to identify barriers towards taking management positions, which could evidently be addressed in management training and development. We could also have asked managers about how they select their successors. Future studies could incorporate these considerations in their research design. Subsequent studies could also compare management trajectories in different countries in order to identify practices that foster and nurture the development of clinical managers across contexts.

### Implications

Firstly, we found that clinicians experienced pressure to enter into management positions. While other studies have suggested that doctors might reluctantly take a management position [24,38], our results suggest that the process by which these individuals engage or “get stuck” [38] in management, could in part be understood as a form of path dependency. To address this issue, it is important to identify and attract motivated clinicians at an early stage. Mountford and Webb [48] have suggested a systematic approach towards gathering and telling stories of successful clinical managers. This might help to increase the pool of interested candidates, so that fewer clinicians become managers by “drawing the short straw” [13].

Secondly, engaging clinicians in management is “about more than simply appointing people to particular positions" [49]. There needs to be a more formalized and structured career path towards management, in which clinicians are offered necessary training and preparation in advance, rather than having to learn “on the fly”. This necessitates a strong organizational interest in management development [50], in which management development “is not a program; it is an organizational commitment” [51]. Lessons can be drawn from organizations that have already fostered successful values and routines for recruiting and developing potential managers. One example is the Mayo Clinic [52], which is recognized for building a culture of organizational support around its managers. Our study suggests that clinical managers would benefit from early advice on how to delegate effectively. Another suggestion would be to offer managers more administrative support in form of designated personnel in assistant or support roles [53-55]. Several authors have suggested the use of mentoring [15,41,56,57]. Nurse managers in Allen´s [15] study noted that social support, often through a mentor, was instrumental in encouraging them to engage in early management experiences. Involving mentors early, and potentially before clinicians enter management, might help to better prepare them for a management role. Creating social arenas and networks for collective sharing of experiences between clinical managers might also prove beneficial.

It is relevant to note that Edmonstone [58] and other authors [59] have criticized the set of assumptions that underlie competency based frameworks, such as in management and leadership development programs in the NHS. Their argument is that competency based approaches may oversimplify management by fragmenting, rather than integrating, different leadership and management activities. Edmonstone and Western [60] state that competency based approaches could prove of limited practical applicability within increasingly complex healthcare organizations, “in which tasks are increasingly complex and messy”. The development of capability, a form of reflective insight derived from practical experience, is argued to be better suited for handling unexpected, ambiguous and dynamic problems.

The distinction between work-based approaches, such as mentoring or coaching, and course-based programs, such as MBAs, could also be relevant when discussing management development. Edmonstone and Western [60] make the point that both types of approaches have their advantages and disadvantages, which need to be recognized in order to move beyond “either/or fashion swings”. For example, while traditional course-based approaches might help to instill important management skills related to HSE and finance, an excessive reliance on externally based programs might be problematic, because of limited time schedules. Edmonstone and Western [60] evaluated two leadership development programs for executive directors of NHS organizations. Participants reported that geographically distant locations were a barrier for attending the programs. On the other hand, skilled mentors might be few in numbers or unavailable locally, which could limit the impact of work-based approaches [61].

Finally, while there is an understanding of the need for appropriate remuneration in private sector organizations, financial incentives for doctors are more often perceived as lacking in public healthcare systems [19]. Chief executives in Ham et als.´ [41] study argued that pay differences could be a major deterrent for experienced hospital specialists who already had significant sources of income from private practice. A lack of appropriate remuneration could also provide frustrated or overworked clinicians with an incentive to opt out of early management roles.

## Conclusion

Path dependency and social pressure seems to influence clinicians’ decisions to enter into management positions. The notion of path dependency is relevant both for theory development and for practical implications. Firstly, the idea of motivated clinician managers needs to be nuanced. While theoretical perspectives from sociological and general management literature emphasize external or internal motivations for engaging in management roles, the path dependency literature provides a framework for understanding other paths into management. In this regard, path dependency might contribute to theory development in the broader area of healthcare organization and management. Secondly, the negative implications of path dependency implies that hospital organizations should formalize pathways into management, in order to identify, attract, and retain the most qualified talents. Newly learned management skills and behaviors also need to be encouraged and supported by the local organization in order to be practiced effectively. If provisional skills learned in external course based programs go unsupported, such courses may be of limited value to clinicians in manager roles. Top managers should consequently make sure that necessary support functions are available locally, especially for early stage clinician managers.

## Competing interests

The authors declare that they have no competing interests.

## Authors’ contributions

All authors were involved in the design of the project. IS carried out the observations and interviews. JCF and LEK provided assistance with coding and analyzing data from the interviews. The drafts of this article were revised critically by all authors. All authors have approved the final version of the manuscript.

## Pre-publication history

The pre-publication history for this paper can be accessed here:

http://www.biomedcentral.com/1472-6963/12/421/prepub
